# ClC-3 chloride channel mediates the role of parathyroid hormone [1-34] on osteogenic differentiation of osteoblasts

**DOI:** 10.1371/journal.pone.0176196

**Published:** 2017-04-24

**Authors:** Xiaolin Lu, Yin Ding, Qiannan Niu, Shijie Xuan, Yan Yang, Yulong Jin, Huan Wang

**Affiliations:** 1 State Key Laboratory of Military Stomatology, Department of Orthodontics, School of Stomatology, The Fourth Military Medical University, Xi’an, Shaanxi, China; 2 Department of Hematology, The Fourth Military Medical University, Xi’an, Shaanxi, China; Universite de Nantes, FRANCE

## Abstract

**Introduction:**

Different concentrations of parathyroid hormone [1–34] (PTH [1–34]) can have totally opposite effects on osteoblasts. Intermittent stimulation with PTH can significantly increase bone mineral density *in vitro*, mainly through the protein kinase A (PKA) signaling pathway, which phosphorylates runt-related transcription factor 2 (Runx2). The ClC-3 chloride channel, an important anion channel, can also promote osteogenesis via the Runx2 pathway based on recent studies. The purpose of our study, therefore, is to research whether the ClC-3 chloride channel has an effect on PTH osteodifferentiation in MC3T3-E1 cells.

**Methods and results:**

A cell counting kit (CCK-8) and real-time PCR were used to investigate the impact of different PTH stimulation modes on MC3T3-E1 cell proliferation and osteogenesis-related gene expression, respectively. We found that the minimum inhibitory concentration of PTH was 10^−9^ M, and the expression of alkaline phosphatase (*Alpl*) and *Runx2* were at the highest levels when treated with 10^−9^ M PTH. Next, we used real-time PCR and immunofluorescence technique to detect changes in ClC-3 in MC3T3-E1 cells under PTH treatment. The results showed higher expression of the ClC-3 chloride channel at 10^−9^ M intermittent PTH administration than in the other groups. Finally, we used the ClC-3 siRNA technique to examine the role of the ClC-3 chloride channel in the effect of PTH on the osteogenesis of osteoblasts, and we found an obvious decrease in the expression of bone sialoprotein (*Ibsp*), osteocalcin (*Bglap*), osterix (*Sp7*), *Alpl* and *Runx2*, the formation of mineralization nodules as well.

**Conclusions:**

From the above data, we conclude that the expression of ClC-3 chloride channels in osteoblasts helps them respond to PTH stimulation, which mediates osteogenic differentiation.

## Introduction

Osteoporosis is a systemic skeletal disease that affects the health and life of millions of people, especially elderly women. It can result in a loss of bone mass and an increased fracture risk [1–3]. Although there are wide variations in treatment practices for osteoporosis, it remains a worsening major public health problem. There are currently two main types of therapy for osteoporosis: those that decrease bone resorption and those that increase bone reformation. Agents that decrease bone resorption, such as bisphosphonates, calcitonin and estrogen, have long been used in clinical practice, albeit with some unwanted disadvantages. Bisphosphonates can lead to gastrointestinal side effects when given orally, such as esophagitis, gastric ulcers and difficulty swallowing [4,5]. Calcitonin is also available to treat osteoporosis, although it is associated with an increased incidence of cancer, hypersensitivity reactions and hypocalcemia [6]. Estrogen is more likely to be associated with the occurrence of breast and endometrial cancer [7]. Attention has therefore gradually begun to shift toward finding a drug that improves bone remodeling.

PTH, an endocrine factor secreted by the parathyroid glands, is the most important hormone regulating bone restructuring *in vivo* [8]. PTH exerts both anabolic and catabolic influences on bone depending on the exposure time [9,10]. Some research has shown that continuous injection of PTH can lead to decreases in bone mass and bone density, while intermittent injection of PTH can significantly increase bone density and bone strength [3,8,11]. These results, in addition to research concerning intermittent concentrations, suggest that PTH has dual effects on osteoblasts under different modes of administration, although the specific mechanisms remain unclear. Furthermore, several signaling pathways are involved in the regulation of bone metabolism [12–15]. The signaling pathways known to mediate the role of PTH in increasing bone quality include cAMP/PKA, IGF1, TGF-β, Runx2 and Wnt, among others [16–20]. Runx2, a specific transcription factor, is closely related to the effect of PTH on osteogenic differentiation [19,20].

The ClC-3 chloride channel is a voltage-gated ion channel that is broadly expressed in mammalian cells and is associated with maintaining cell volume balance, regulating cell excitability, ion homeostasis, lysosomal acidification and transmembrane transportation [21–23]. Recent studies have also found that the ClC-3 chloride channels expressed in osteocytes not only participate in cell proliferation and apoptosis of osteoclasts [24,25], but also promote new bone growth via osteoblasts. According to the latest research, the ClC-3 chloride channel is strongly expressed in murine osteoblast lineage cells, which plays an important role in enhancing the mineralization ability of osteoblasts *in vitro* and in promoting osteogenic differentiation [26,27], but also exists in mineralizing osteoblasts in humans, which it is expressed at a higher level than in growing cells [28]. The Overexpression of ClC-3 chloride channel could enhance the expression of osteogenic markers (*Alpl*, *Ibsp* and *Bglap*) and contribute to the calcification ability of MC3T3-E1 cells, whereas knockdown of ClC-3 chloride channel has the opposite result. Similarly, studies also showed that ClC-3 chloride channels regulate osteogenesis behavior through the Runx2 transcription factor [27,29].

Because both PTH and ClC-3 chloride channels can promote osteogenesis in bone metabolism and may have similar mechanisms via regulating osteogenesis, it is important to research whether there are internal links between these two elements in osteogenic differentiation. Research on this topic has not been reported until now. Therefore, in the current study, we aimed to investigate whether ClC-3 chloride channels mediate the role of PTH on osteogenic differentiation in osteoblasts.

## Materials and methods

### Cell culture and PTH stimulation

Murine pre-osteoblastic MC3T3-E1 cells were purchased from American Type Culture Collection (ATCC) agency (Shanghai, China), and cultured in α-MEM (Gibco, USA) with 10% fetal bovine serum (FBS) (Gibco, USA) and 1% penicillin-streptomycin within a humidified incubator at 37°C containing 5% CO_2_. Synthesized human PTH (1–34) (Sigma USA) was dissolved in PBS (phosphate buffered saline) to a concentration of 1.21 × 10^− 4^ M (stock solution) and then dissolved to a final concentration of 10^− 8^ M, 10^− 9^ M and 10^− 10^ M.

MC3T3-E1 cells were trypsinized and plated at a density of 2 × 10^−4^ cells/well in 6-well plates and grown to 70% confluency for PTH stimulation. The cells were divided into three groups: the continuous, intermittent and control groups. The continuous group was treated with PTH at 10^− 8^ M, 10^− 9^ M and 10^− 10^ M for 24 h. The intermittent group was exposed to PTH of different concentrations for the first 6 h and then replaced with culture medium free from PTH during the remainder of the cycle. The control group was cultured with common medium supplemented with 10% FBS only. Culture media of all groups were changed every 24 h (one cycle). This experiment consisted of three cycles.

### Cell proliferation assay

A total of 100 μl of cell suspension (1×10^4^ cells/well) was seeded in 96-well microplates, and the plates were incubated in a humidified environment (37°C, 5% CO_2_). Different groups were stimulated with PTH. After the cells adhered, 10 μl of CCK-8 (Dojindo, Tokyo, Japan) solution was added to each well. After 4 h, we used a microplate reader (Beckman-Coulter, CA, USA) to detect the absorbance at 450 nm.

### ClC-3 siRNA and gene transfection

A small interfering RNA (siRNA) targeting the mouse ClC-3 was synthesized by GenePharma (Shanghai, China) according to previously described methods. The siRNA sequences were as follows: sense: 5’-CGA GAG AAG UGU AAG GAC ATT-3’; antisense: 5’-UGU CCU UAC ACU UCU CUC GTT-3’. The nonsense siRNA sequences were sense, 5’-UUC UCC GAA CGU GUC ACG UTT-3’, and antisense: 5’-ACG UGA CAC GUU CGG AGA ATT-3’. Before gene transfection, the cells were seeded in six-well plates (1 × 10^4^ cells/well) and treated with PTH. Then, the cells were transfected with siRNA duplexes using Lipofectamine 2000 (Lipofectamine^™^2000, Invitrogen, USA) per the manufacturer’s instructions. The regular medium was refreshed after 6 h, and the cells were continuously cultured for 48 h for the next step.

### Real-time PCR assay

The total RNA of the cells was isolated using an E.Z.N.A^®^Total RNA Kit (Omega, USA) according to the manufacturer’s protocols. Next, the RNA was reverse-transcribed with PrimeScript^®^ RT Master (TaKaRa, Dalian, China) to cDNA. Then, the real-time PCR reaction was conducted using the ABI 7500 Real-Time PCR System with Premix (TaKaRa, Dalian, China). The thermocycling reaction was performed at 95°C for 30 s for initial denaturation, then 45 cycles of denaturation at 95°C for 5 s, and finally annealing at 60°C for 34 s. The primer sequences used are shown in Table 1. The comparative threshold cycle (ΔΔCT) method was used to quantify gene expression, and we elected to use Gapdh as the internal control.

**Table 1:** Primer sequences and expected sizes of PCR products.

Gene	Forward primer	Reverse primer	Size (bp)
Alpl	5’-CCAACTCTTTTGTGCCAGAGA-3’	5’-GGCTACATTGGTGTTGAGCTTTT-3’	110
Ibsp	5’-CAGGGAGGCAGTGACTCTTC-3’	5’-AGTGTGGAAAGTGTGGCGTT-3’	158
Clcn3	5’-CCAAGACCCCGCTTCAATAA-3’	5’-CGAGTCCCGCAGATTAAAGA-3’	112
Bglap	5’-CTGACCTCACAGATCCCAAGC-3’	5’-TGGTCTGATAGCTCGTCACAAG-3’	187
Sp7	5’-ATGGCGTCCTCTCTGCTTG-3’	5’TGAAAGGTCAGCGTATGGCTT-3’	156
Runx2	5’-CGCCCCTCCCTGAACTCT-3’	5’-TGCCTGCCTGGGATCTGTA-3’	72
Gapdh	5’-CATGTTCCAGTATGACTCCACTC-3’	5’-GGCCTCACCCCATTTGATGT-3’	136

### Immunofluorescence analysis

MC3T3-E1 cells were plated in culture dishes specialized for immunofluorescence at a density of 1 × 10^4^/ml. PTH was administered as described above. Next, the cells were fixed with ice cold 4% paraformaldehyde for 20 min, permeabilized with 0.25% Triton X-100 for 5 min, and blocked with 3% bovine serum albumin for 30 min at 37°C. The cells were then incubated with primary antibodies (CST, USA) overnight at 4°C, followed by secondary conjugated antibodies (CST, USA) for 45 min at 37°C after several rinses with PBS. The images were obtained using a laser scanning confocal microscope (FV1000 System, Olympus, Japan).

### Alizarin Red S staining

To evaluate the mineralization level of MC3T3-E1 cells, Alizarin Red S Staining was performed in the study. The transfection of ClC-3 siRNA was repeated six times every 3 days over a period of 21 days to evaluate the long-term effect. After 21 days, the cultured cells were fixed in 4% paraformaldehyde for 20 min and then stained with Alizarin Red S (2% aqueous, pH 4.2, Sigma) for 30 min. The images were observed using an inverted phase contrast microscope, and the quantification of the staining based on the Alizarin Red S standard was determined by measuring the absorbance at 562 nm.

### Statistical analysis

All data were analyzed using SPSS statistical software (Version 17.0; SPSS, Chicago, IL, USA) and expressed as the means ± standard deviation (SD). The statistics were executed using Prism GraphPad (version 6.00) for Windows. The Student’s t-test was used to detect the difference between two groups. Comparisons between three or more groups were analyzed by one-way analysis of variance (ANOVA) followed by the Least Significant Difference (LSD) test and Student-Newman-Keuls (SNK) q test. P values less than 0.05 were considered significantly different. All experiments were repeated more than three times.

## Results

### Effects of PTH stimulation on proliferation of MC3T3-E1 cells

**Fig 1:**
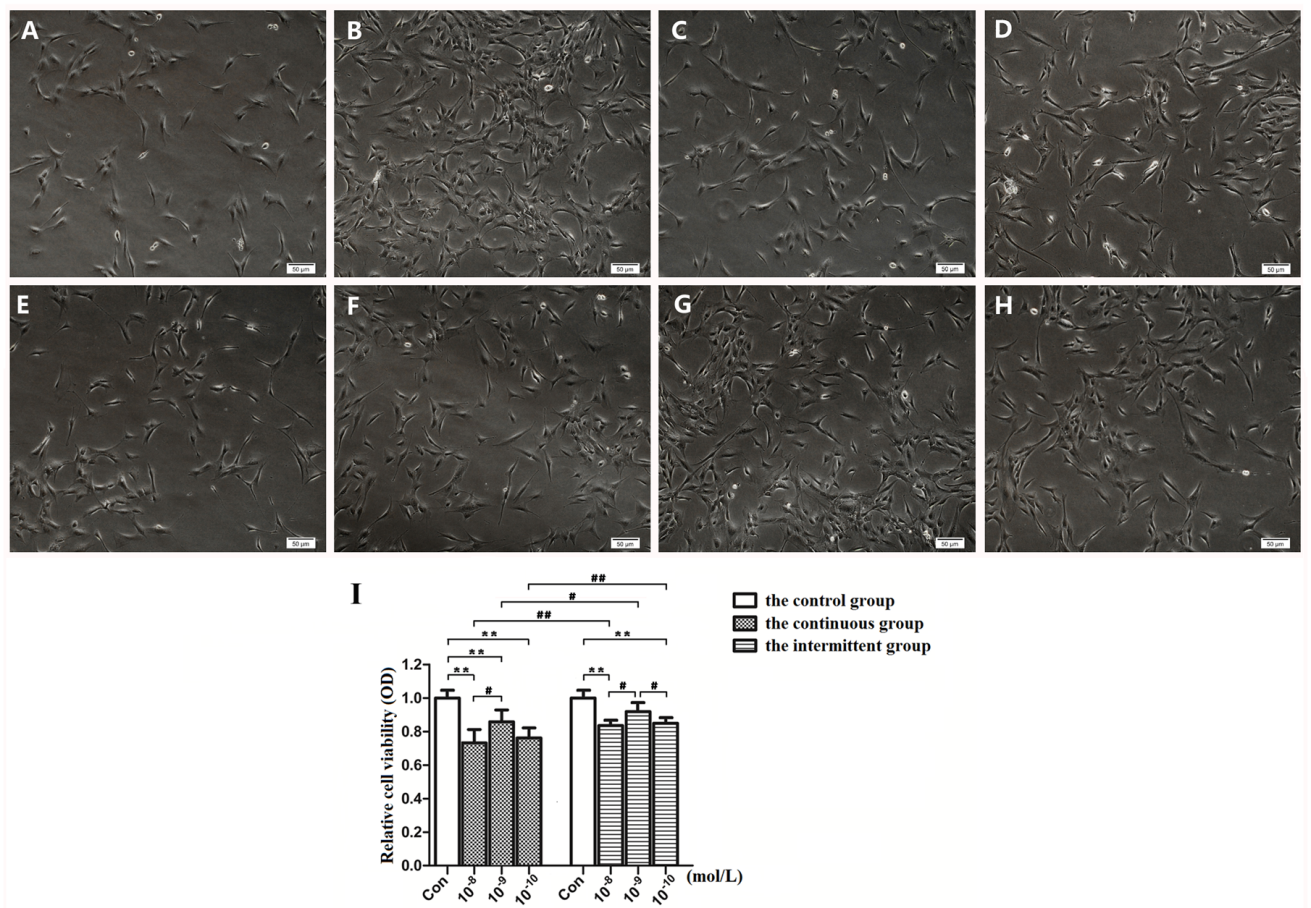
Effects of different PTH stimulations on proliferation of the MC3T3-E1 cells. (A-H) The morphology of cells before and after different PTH stimulation modes. (A) The control group; (B) The control group at 72 h; (C), (D) and (E) The continuous group treated with 10^−8^, 10^−9^, and 10^−10^ M PTH; (F), (G) and (H) The intermittent group treated with 10^−8^, 10^−9^, and 10^−10^ M PTH. (I) The quantification of live cells in each group. To quantify the number of cells, the optical density (OD) value was used to detect the cell proliferation. The results showed that the minimum inhibitory concentration of PTH was 10^−9^ M. Scale bars = 50 μm. *P<0.05, **P<0.01 compared with the control group. #P<0.05, ##P<0.01 compared between the experimental groups.

The cellular morphological changes observed under an inverted phase contrast microscope showed that the cells in each group were substantially the same, with fusiform cytoplasm and long, thin cell processes (Fig 1A–1H). The CCK-8 assay showed that both continuous and intermittent administration of PTH inhibited the proliferation ability of MC3T3-E1 cells compared with the control group. Additionally, the intermittent PTH stimulation groups showed less inhibition than the continuous stimulation groups at the same PTH concentrations. Of note, 10^−9^ M PTH appeared to be the minimum inhibitory concentration for promoting cell proliferation (Fig 1I).

### Osteogenic gene expression profiles in MC3T3-E1 cells under PTH stimulation

**Fig 2:**
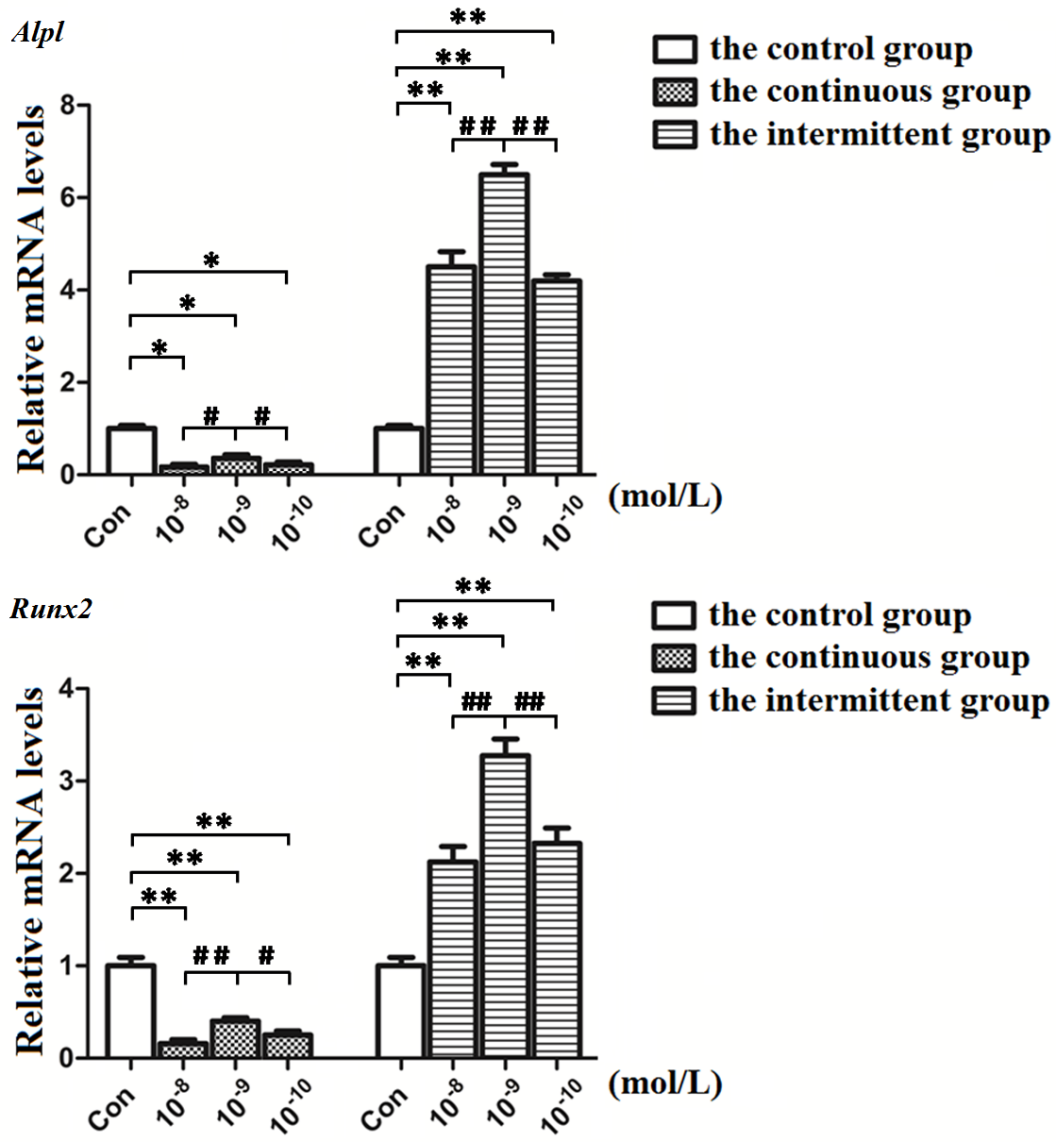
Effects of different PTH administration modes on the mRNA expression of *Alpl* and *Runx2*. Expression of *Alpl* and *Runx2* was decreased in the continuous groups and increased in the intermittent groups. In the intermittent groups, the genes were significantly differently expressed at 10^−9^ M PTH. *P<0.05, **P<0.01 compared with the control group. #P<0.05, ##P<0.01 compared between the experimental groups.

We then detected the expression of *Alpl* and *Runx2* to evaluate the influence of different concentrations and treatments of PTH on osteogenic genes. The real-time PCR results showed that continuous PTH stimulation suppressed *Alpl* and *Runx2* expression. On the contrary, intermittent PTH stimulation can increase the mRNA expression levels of the above genes. At the concentrations of 10^−8^ M, 10^−9^ M and 10^−10^ M, 10^−9^ M PTH significantly promoted gene expression. In summary, these findings indicated that intermittent PTH at 10^−9^ M was the most effective administration concentration to promote osteogenic differentiation (Fig 2).

### Effects of PTH stimulation on ClC-3 expression in MC3T3-E1 cells

**Fig 3:**
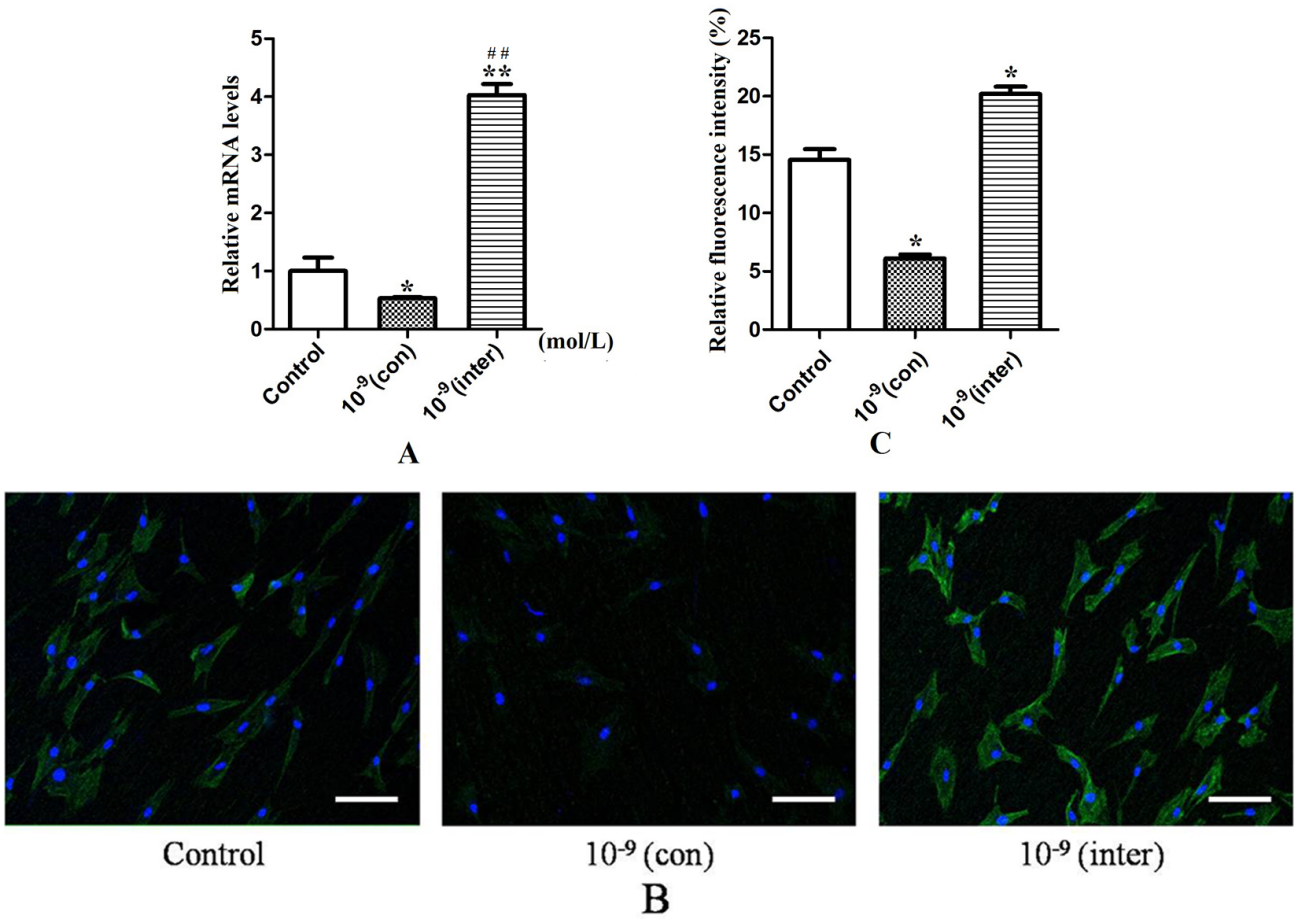
The effect of 10^−9^ M PTH on ClC-3 chloride channel expression. (A) The mRNA expression of *Clcn3*; (B) The immunofluorescence expression of ClC-3 protein; (C) Quantification of fluorescence of ClC-3. The ClC-3 protein was expressed intensively at 10^−9^ M PTH via intermittent stimulation, while it was only weakly expressed at 10^−9^ M PTH via continuous stimulation. Control: the control group; 10^−9^(con): the group treated with 10^−9^ M PTH continuously for 72 hours; 10^−9^(inter): the group treated with 10^−9^ M PTH intermittently for 72 hours. Scale bars = 50 μm. *P<0.05, **P<0.01 compared with the control group. ##P<0.01 compared with the 10^−9^(con) group.

To monitor the change in ClC-3 chloride channels under 10^−9^ M PTH stimulation, we first used real-time PCR to detect the mRNA levels of *Clcn3* (Fig 3A). The results showed that continuous PTH treatment at 10^−9^ M inhibited *Clcn3* expression, whereas intermittent PTH stimulation at 10^−9^ M elevated the mRNA of *Clcn3* when compared with the control group. We next examined the expression of ClC-3 protein using immunofluorescence techniques (Fig 3B), and the images revealed that the ClC-3 protein exhibited stronger coloring in MC3T3-E1 cells in the intermittent group (Fig 3C) than the cells in the continuous group. The control group had coloring that was in between that of the intermittent and continuous groups (Fig 3C).

### PTH promotion of osteogenic gene expression in MC3T3-E1 cells through ClC-3

**Fig 4:**
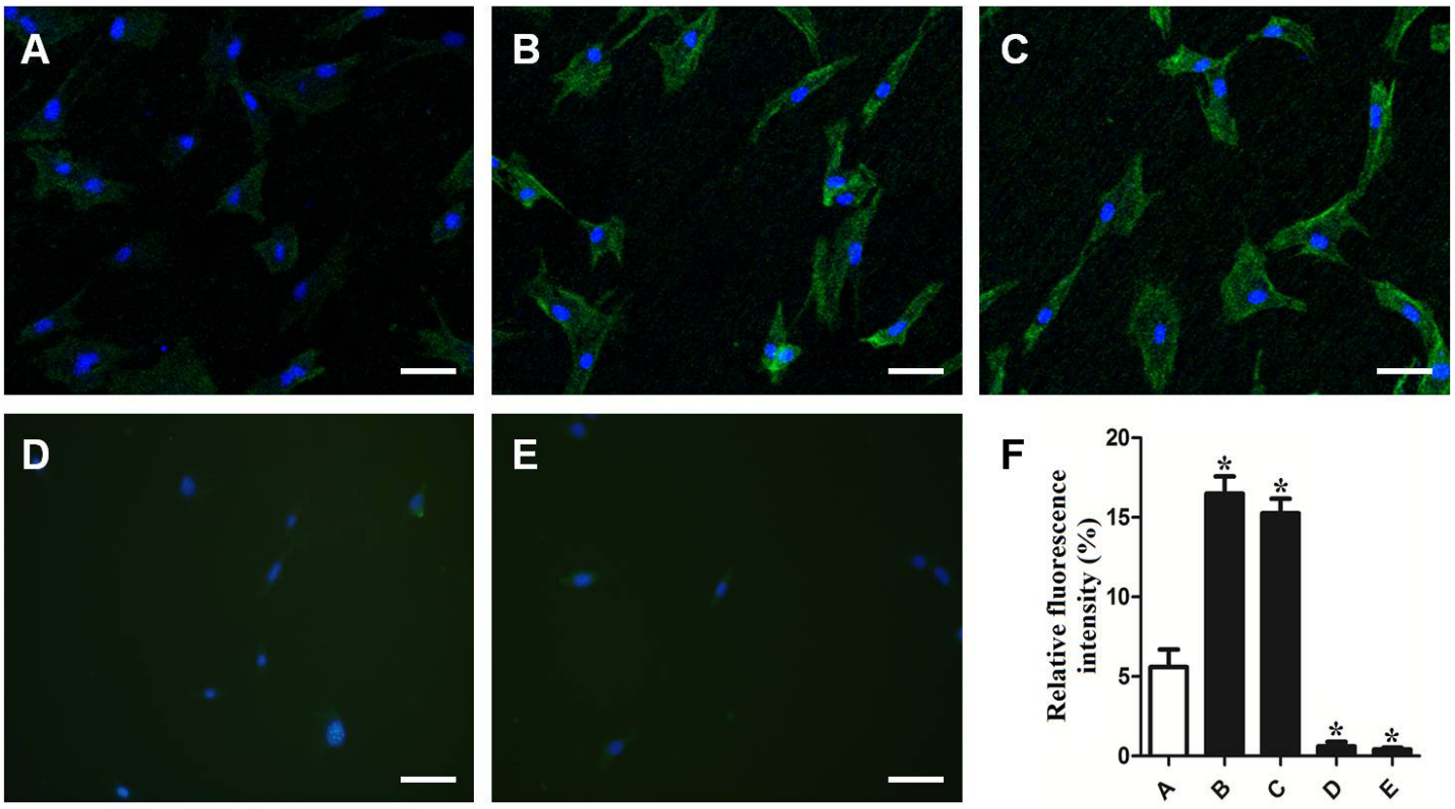
Effects of different ClC-3 chloride channel expression levels on ClC-3 protein expression. (A) The control group; (B) 10^−9^ M intermittent PTH stimulation group; (C) 10^−9^ M intermittent PTH stimulation and nonsense siRNA transfection group; (D) 10^−9^ M intermittent PTH stimulation and ClC-3 siRNA transfection group; (E) ClC-3 siRNA transfection group; (F) Quantification of ClC-3 fluorescence among the five groups. The protein expression of ClC-3 was promoted following treatment with 10^−9^ M PTH, and inhibited following ClC-3 siRNA transfection with or without 10^−9^ M intermittent PTH stimulation. Scale bars = 50 μm. *P<0.05 compared with the control group.

**Fig 5:**
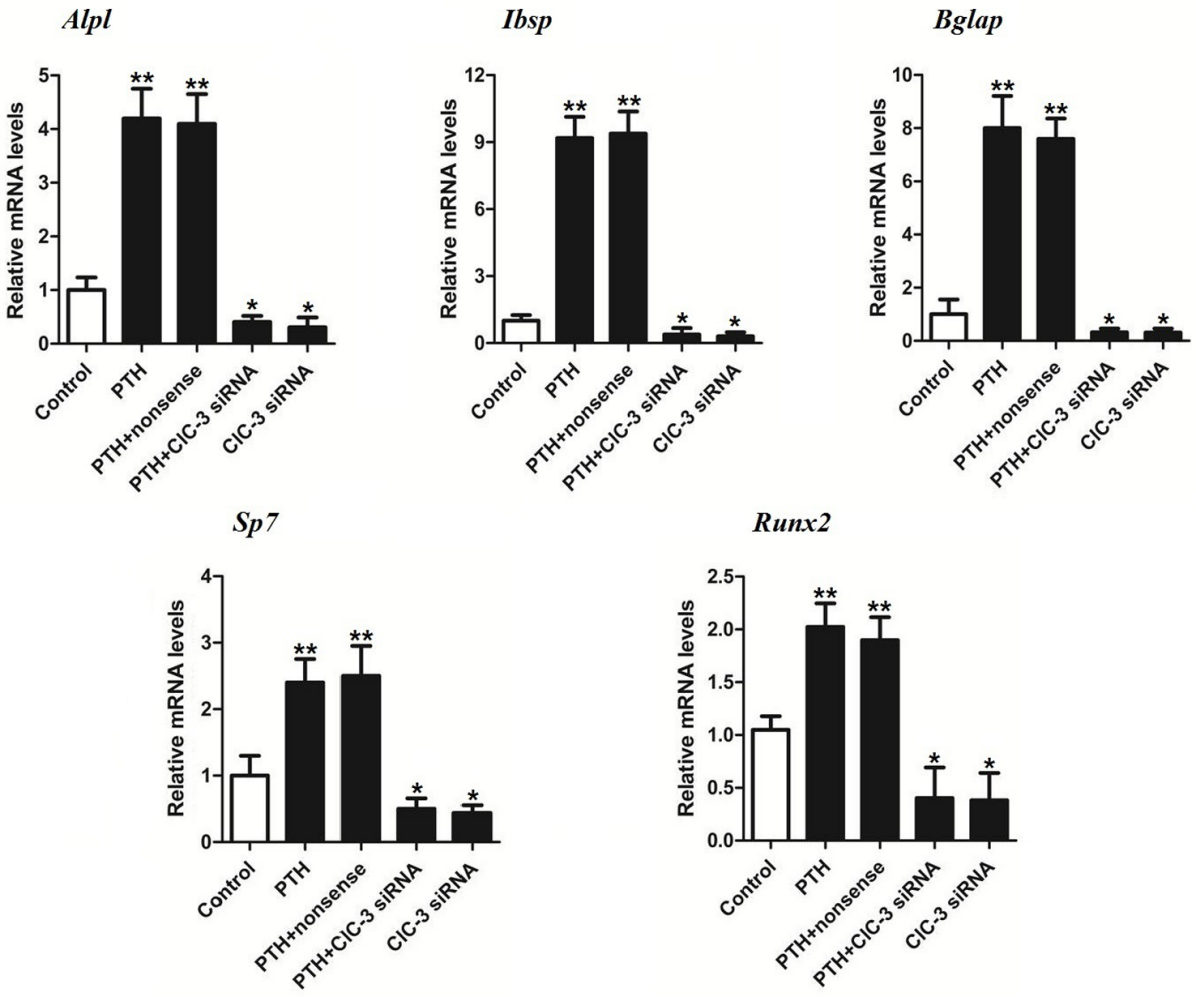
Effects of different ClC-3 chloride channel expression levels on osteogenesis-related genes. Control: the control group; PTH: the cells treated only with intermittent PTH stimulation at 10^−9^ M; PTH+nonsense siRNA: the cells treated with intermittent PTH stimulation at 10^−9^ M and then transfected with nonsense siRNA; PTH+ClC-3 siRNA: the cells treated with intermittent PTH stimulation at 10^−9^ M and then transfected with ClC-3 siRNA; ClC-3 siRNA: the cells transfected with ClC-3 siRNA. The gene expression levels (*Alpl*, *Ibsp*, *Bglap*, *Sp7* and *Runx2*) were increased after stimulation with 10^−9^ M PTH, and the gene expression levels were decreased in PTH+ClC-3 siRNA and ClC-3 siRNA groups. *P<0.05, **P<0.01 compared to the control group.

**Fig 6:**
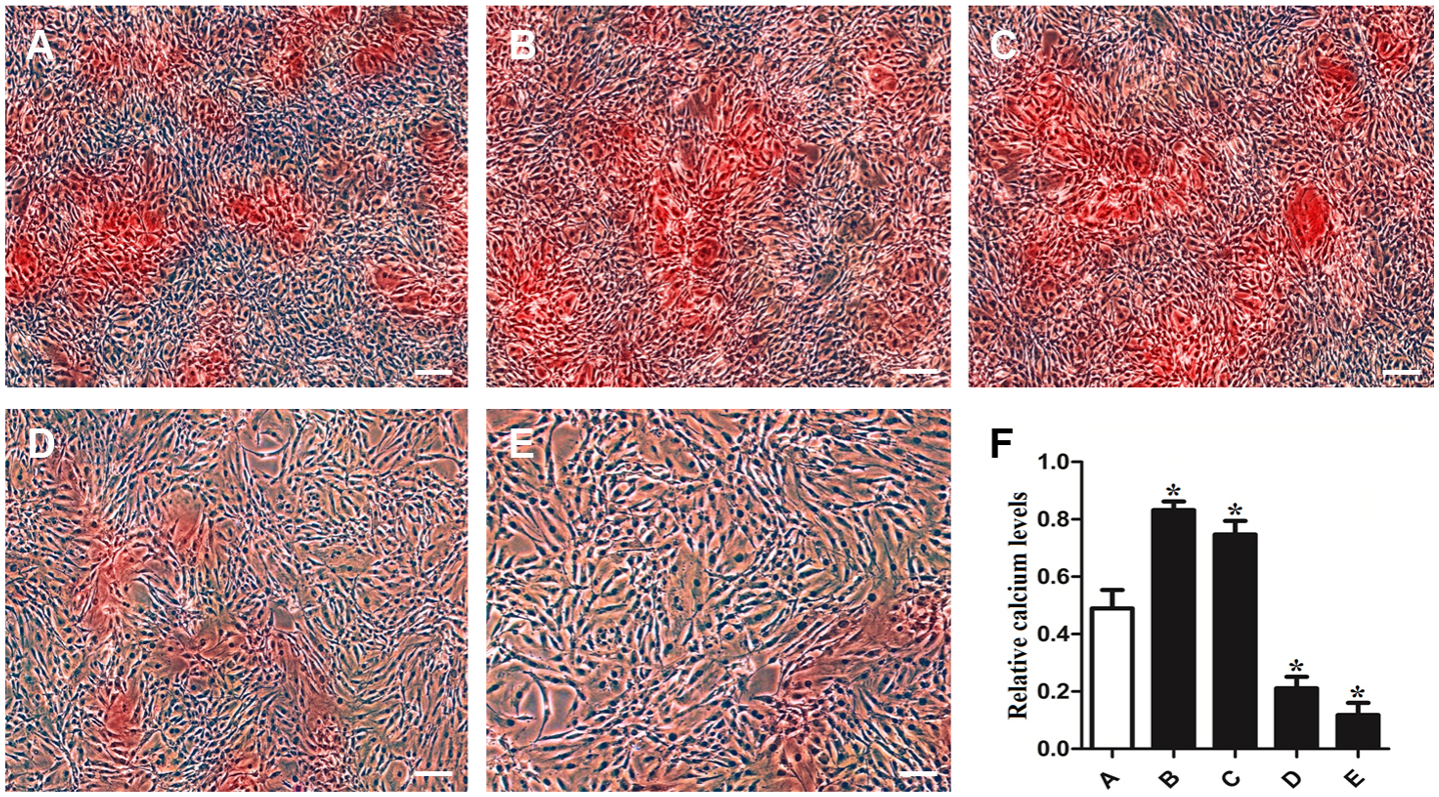
Effects of different ClC-3 chloride channel expression levels on mineralization. (A) The control group; (B) 10^−9^ M intermittent PTH stimulation group; (C) 10^−9^ M intermittent PTH stimulation and nonsense siRNA transfection group; (D) 10^−9^ M intermittent PTH stimulation and ClC-3 siRNA transfection group; (E) ClC-3 siRNA transfection group; (F) Quantification of calcium deposition. The Alizarin Red S positive staining area in group (B) and group (C) were more intensive than in the control group, whereas the staining area were less intensive in group (D) and group (E) than in the control group. Scale bars = 50 μm. *P<0.05 compared to the control group.

To further investigate the role of ClC-3 in regulating the expression of osteogenic genes, MC3T3-E1 cells were intermittently stimulated with 10^−9^ M PTH. We mainly used siRNAs to interfere with the ClC-3 chloride channels, and we divided the sample into five groups: ClC-3 siRNA, PTH+ClC-3 siRNA, PTH+nonsense, PTH and a control group. The immunofluorescence analysis indicated that the fluorescence in the PTH-treated cells was more intense than that in the ClC-3 gene-silenced cells (Fig 4). The real-time PCR data showed that transfection with ClC-3 siRNA with or without 10^−9^ M intermittent PTH stimulation reduced the expression of *Alpl*, *Ibsp*, *Bglap*, *Sp7* and *Runx2*, whereas transfection with nonsense and PTH stimulation enhanced gene expression compared to the control group (Fig 5). Based on these data, we found that PTH could up-regulate osteogenic gene expression through ClC-3 chloride channels. Furthermore, we measured calcium deposition in MC3T3-E1 cells via optical density to determine the capacity of mineralization. Alizarin Red S staining at 21 days demonstrated that calcium nodules were significantly increased in the PTH treatment group compared to the control group, whereas the calcium deposition was minimal in the ClC-3 siRNA group (Fig 6). Taken together, PTH promoted osteogenic differentiation in MC3T3-E1 cells through the ClC-3 chloride channel, and knockdown of ClC-3 significantly reduced the osteogenesis effect of PTH.

## Discussion

In this study, we demonstrated the dual effects of different PTH administrations protocols in MC3T3-E1 cells, and determined the optimal PTH concentration. We further detected that PTH preferentially activates the ClC-3 chloride channel to regulate the expression of osteogenesis-related genes and calcium nodules using a mineralization test. This may be the first report to show that ClC-3 channels mediate the role of PTH on osteogenic differentiation.

Bone is a type of tissue that is constantly being broken down and rebuilt throughout the lifetime. Bone metabolism regulated by local factors and endocrine hormones involved in body growth. Any disorder of the process leads to abnormal bone formation. Osteoporosis is a condition in which bone resorption is faster than fresh bone creation [30,31]. The treatment of osteoporosis via inhibiting bone resorption has some limitations. Therefore, an increasing amount of research has focused on bone rebuilding. PTH, one of the most important hormones in regulating bone metabolism, plays an important role in maintaining body homeostasis. Interestingly, PTH appears to exert diverse effects. Continuous PTH treatment decreases bone mass, while pulsatile stimulation enhances bone mineral density. The anabolic influence of intermittent PTH injection works mainly by stimulating preosteoblast proliferation, promoting preosteoblast and osteoblast differentiation and inhibiting osteoblast apoptosis. Previous studies [9] have focused mainly on the different outcomes of continuous and discontinuous PTH *in vitro*, and they reached the conclusion that PTH promoted bone formation by activating multiple signaling pathways, such as cAMP/PKA, IGF1, TGF-β, Runx2 and Wnt [16–20]; however the particular mode of administration and concentration of osteoblasts were not explicit. These studies mostly used 10^−7^ M or 10^−8^ M PTH for the pulsatile intervention concentration to observe the expression of osteogenesis-related genes under different periods of each cycle [10,32]. However, there are few studies describing the dose of PTH most effective in promoting bone formation. Therefore, in this study, we aimed to select the PTH concentration that best promoted osteogenesis. After a series of pre-tests, we finally chose 10^−8^ M, 10^−9^ M and 10^−10^ M as the experimental concentrations. A CCK-8 assay is used to measure cell proliferation, and we found that all levels of PTH inhibited MC3T3-E1 cell proliferation compared with the control group. Additionally, the intermittent pattern resulted in lower inhibition than continuous administration, and the concentration 10^−9^ M showed the lowest inhibition among the three doses. Some studies have demonstrated that PTH enhances the differentiation of bone marrow mesenchymal stem cells (BMSCs) and chondrocytes [33,34]. Therefore, intermittent PTH treatment likely promoted bone formation in MC3T3-E1 cells through cell differentiation but not proliferation, which requires verification through further studies. Osteoblastic differentiation is a crucial process in bone formation and is regulated by multiple factors. The maturation of pre-osteoblasts is distinguished by changes in bone surface markers. The activity of *Alpl*, one of the early differentiation markers of osteoblasts [35], significantly induces osteogenic differentiation. *Runx2*, a critical central regulation factor of osteoblast differentiation, is affected by upstream factor regulation and induces the expression of downstream target genes. Therefore, we assessed the mRNA expression of *Alpl* and *Runx2* to determine the optimal concentration of PTH for osteogenic differentiation by using 10^−8^ M, 10^−9^ M and 10^−10^ M PTH to stimulate MC3T3-E1 cells continuously and intermittently in the study. The observed effects on gene expression in the C-PTH and I-PTH groups were opposite, suggesting differential regulation of osteo-differentiation by the various PTH modes. In the 10^−9^ M intermittent treatment group, *Alpl* and *Runx2* were significantly elevated compared with the other groups, which is partly consistent with the finding that *Runx2* expression is dependent on the concentration of PTH [36]. Thus, this finding suggests that the effects of different PTH concentrations on the expression of osteogenesis genes varies, demonstrating a parabolic shape. Therefore, we can conclude that intermittent stimulation of MC3T3-E1 cells with 10^−9^ M PTH is the optimal administration concentration for bone promotion.

The ClC-3 chloride channel is an important anion channel in living creatures. In addition to having well-known functions, our recent research revealed that ClC-3 has a close relationship with bone metabolic processes and regulates osteoblast proliferation, differentiation and apoptosis to maintain the balance of bone remodeling [21,22]. *Clcn3* knockout mice were characterized by developmental retardation and spinal deformity after birth, and osteoclasts isolated from *Clcn3* knockout mice showed lower bone absorption [37]. More importantly, studies have shown that ClC-3 chloride channels can increase the expression of osteogenesis-related genes and mineralized nodule formation in osteoblasts by regulating the Runx2 transcription factor [27]. Interestingly, the effect of PTH on promoting bone formation is closely related to the Runx2 pathway [19,20]. Here, we hypothesized that the ClC-3 chloride channel may mediate the role of PTH in osteogenic differentiation in osteoblasts.

To explore the correlation between PTH and the ClC-3 chloride channel, we firstly utilized real-time PCR to observe *Clcn3* expression following different PTH administration protocols. The data showed that the gene expression level of *Clcn3* was the highest after discontinuous treatment of MC3T3-E1 cells with 10^−9^ M PTH, and the intensity of the fluorescence staining was significantly higher in the same condition. This result indicated that the expression of ClC-3 chloride channel was prominent when MC3T3-E1 cells were treated with 10^−9^ M PTH intermittently. Some studies [29,38] have shown ClC-3 can promotes osteogenic differentiation in MC3T3-E1 cells after mechanical stimulation. In this study, we can assume that PTH may have acted as a kind of physiological stimulation, thereby activating the expression of ClC-3 chloride channels, and accelerating the expression of osteogenic-related genes. However, whether ClC-3 mediated the role of PTH in osteogenic differentiation through Runx2 gene signaling pathway, further research is needed to determine the specific mechanism.

To test the further relationship between PTH and ClC-3 in osteogenesis, we down-regulated the expression of ClC-3 chloride channels to view the change in osteoblastic genes and mineralization. In our study, we mainly used siRNA, one mode of gene transfection, to knockdown ClC-3 chloride channels, and the effectiveness of the ClC-3 siRNA sequence has been verified [26]. We found that the expression of ClC-3 protein upon transfection with ClC-3 siRNA was inhibited, whereas the expression of ClC-3 protein in cells treated with 10^−9^ M PTH was promoted. This result further suggested that ClC-3 expression is activated by PTH. Studies have suggested that ClC-3 chloride channels may exist in either an active dephosphorylated state or a closed phosphorylated state [39]. ClC-3 channels become dephosphorylated and more channels open when PKC activity is diminished under hypotonic conditions [40]. PTH can also rapidly stimulated PKC activity within 30 sec in renal tissue but declines rapidly thereafter, whereas the duration of PKA activation is much longer than PKC [41]. Thus, we can assume that PTH activated ClC-3 chloride channels after PKC activity diminished. Bone sialoprotein plays an important role in the initial differentiation of osteoblasts and bone matrix mineralization [42]. Osteocalcin, possibly the most common protein used to create a good locator for hydroxyapatite, ultimately offers a good substrate for new bone formation [43]. Osterix is also required for osteoblastic differentiation and acts as a downstream factor of Runx2 [44]. In our study, we detected the expression of osteogenesis-related genes after we blocked the expression of ClC-3 chloride channels. In the group treated with 10^−9^ M PTH and ClC-3 siRNA, the expression of *Alpl*, *Ibsp*, *Bglap*, *Sp7* and *Runx2* were inhibited. However, the group stimulated with the appropriate concentration of PTH displayed increased mRNA expression of these genes, which is consistent with the findings of previous studies [45]. As mentioned above, *Ibsp*, *Sp7* and *Runx2* are early osteoblastic markers in preosteoblastic cells, whereas *Bglap* is late marker [46]. Among the above genes, *Ibsp* was expressed in ClC-3 siRNA group at a level 1/20 as high as that in PTH group, whereas the other genes (*Alpl*, *Sp7* and *Runx2*) nearly had 1/10 expression levels. This finding indicated that ClC-3 chloride channels may have an effect in the early period of the bone reestablishment process. Mineral nodule formation is one of the signs of bone formation in cell culture system *in vitro*. The results in our study showed that the optimal administration of PTH increased the number of mineral nodules, whereas knockdown of ClC-3 channels reduced calcium nodule formation. This finding suggests that ClC-3 may affect the function of PTH in mineralization. Although ClC-3 itself impacts osteogenic differentiation and mineralization in preosteoblasts [21,22], when we treated MC3T3-E1 cells with 10^−9^ M PTH intermittently, we observed higher expression of ClC-3 channels, as well the osteogenesis-related genes and mineral nodules. Blockade of ClC-3 chloride channel markedly reduced the expression of osteogenesis-related genes. Previous studies demonstrated that ClC-3 chloride channels can promote the distribution of certain related genes in osteoblasts by regulating the Runx2 transcription factor [27]. The expression of Runx2 was significantly upregulated after ClC-3 overexpression in MC3T3-E1 cells. After mRNA knockdown of Runx2, ClC-3-regulated osteo-differentiation was inhibited. Meanwhile, the activity of Runx2 is essential to mediate the osteoblastic effects of PTH. PTH also suppresses osteoblast apoptosis by phosphorylating PKA and inactivating the pro-apoptotic protein Bad via activation of the cAMP and PKA pathways. It has been suggested that Runx2 and cAMP response element-binding protein (CREB) may be the key mediators of anti-apoptotic effect of PTH in osteoblast [47,48]. Krishnan also discovered that the expression and activity of Runx2 was elevated by intermittent PTH stimulation, which promoted preosteoblasts transformation into osteoblasts [36]. Thus, PTH and ClC-3 chloride channels may both play a part in the regulation of bone metabolism via the Runx2 signaling pathway. This phenomenon provides a new direction to gain insight into the mechanism underlying bone formation and lays the foundation for PTH research *in vivo*, which will be beneficial in the treatment of osteoporosis. Furthermore, the complex network of the signaling pathways still requires deeper exploration.

## Conclusions

In conclusion, the optimal PTH administration approach for promoting osteoblast and osteogenic differentiation is intermittent stimulation at a concentration of 10^−9^ M. The expression of ClC-3 chloride channels in osteoblasts can respond to the stimulation with PTH and serve as a mediator when PTH promotes osteogenic differentiation in osteoblasts.
